# Knockdown of Bach1 protects periodontal bone regeneration from inflammatory damage

**DOI:** 10.1111/jcmm.17916

**Published:** 2023-08-21

**Authors:** Zhiyao Yuan, Junjie Li, Xihong Zou, Chaoyi Liu, Jiangyue Lu, Can Ni, Lai Tang, Xudong Wu, Fuhua Yan

**Affiliations:** ^1^ Department of Periodontology, Nanjing Stomatological Hospital, Affiliated Hospital of Medical School Nanjing University Nanjing China; ^2^ State Key Laboratory of Pharmaceutical Biotechnology, School of Life Sciences Nanjing University Nanjing China; ^3^ Hangzhou Stomatological Hospital Hangzhou China

**Keywords:** Bach1, osteogenesis, oxidative stress, periodontal ligament cells (PDLCs), periodontitis

## Abstract

Periodontal bone regeneration is a major challenge in the treatment of periodontitis. However, the regenerative vitality of periodontal ligament cells (PDLCs) declines in the environment of periodontitis and accompanying oxidative stress. This study aimed to investigate the functional mechanisms of Bach1, a transcriptional suppressor involved in oxidative stress response, and its regulation of PDLC osteogenesis under inflammatory conditions. We observed a significant elevation in Bach1 expression in periodontal tissues with periodontitis and PDLCs under inflammatory conditions. Knockdown of Bach1 alleviated the inflammation‐induced oxidative stress level and partly offset the inhibitory effect of inflammatory conditions on osteogenesis, as well as the expression of osteogenic genes BMP6, OPG and RUNX2. Similarly, knockdown of Bach1 protects PDLCs from inflammatory damage to periodontal bone regeneration in vivo. Furthermore, we found that Bach1 could bind to the histone methyltransferase EZH2, and the binding increased under inflammatory conditions. Bach1 enhanced the ability of EZH2 to catalyse H3K27me3 on the promoter region of RUNX2 and BMP6, thus repressing the expression of osteoblastic genes. In conclusion, our study revealed that knockdown of Bach1 effectively rescued the osteogenesis and oxidative stress of PDLCs with inflammation. Bach1 could be a promising target for enhancing periodontal tissue regeneration under periodontitis conditions.

## 1 INTRODUCTION

Periodontitis is a chronic infectious disease of the periodontal support tissue caused by microbial plaque, which leads to periodontal support tissue destruction, progressive loss of attachment and alveolar bone absorption.^1^, ^2^ Periodontitis is one of the most common oral diseases in adults and the main cause of tooth loss in adults.^3^, ^4^ At present, part of the plaque can be removed, and inflammation can be controlled by periodontal debridement therapy, such as scaling and flap surgery, but it is difficult to achieve the regeneration of periodontal tissue, including periodontal ligament (PDL), cementum and alveolar bone.^5^, ^6^ Periodontal ligament cells (PDLCs) are a group of heterogeneous cells in periodontal tissue with certain characteristics of stem cells and can differentiate into PDL, alveolar bone and cementum.^7^, ^8^ Hence, PDLCs are considered the major cells of natural periodontal tissue regeneration and repair. However, when PDLCs are used to repair periodontal bone defects, the inflammatory microenvironment affects the biological function of PDLCs, resulting in decreased regenerative potential.^9^ Inflammatory factors, including TNF‐α and IL‐1β, are involved in the development of periodontitis and induce reactive oxygen species (ROS) production and oxidative damage in periodontal tissues. People who are hyperreactive to ROS are more susceptible to developing periodontitis than those who have a normal ROS response.^10^ Therefore, preventing PDLCs from oxidative injury and the consequent reduction in osteogenesis could represent an effective therapeutic strategy for periodontitis treatment.

BTB‐CNC homology 1 (Bach1) is a transcriptional suppressor involved in the regulation of mitochondrial metabolism, oxidative stress response, tumour metastasis, cell cycle regulation and self‐renewal of stem cells.^11^, ^12^, ^13^, ^14^ The expression level of Bach1 was increased in ageing tissues with ageing damage caused by long‐term oxidative stress and inflammatory stimulation.^15^, ^16^ Bach1 competes with Nrf2 for the antioxidant response element (ARE), subsequently inhibiting the expression of many antioxidant factors, including haem oxygenase‐1 (HO‐1), which is a key factor in antioxidative stress, antiapoptotic and anti‐inflammatory processes to activate cell protection, as well as other oxidative stress response genes, such as Gclm, Gclc, Fth1 and Ftl1.^12^, ^17^, ^18^ Hence, genetic ablation of Bach1 could suppress ferroptosis and relieve the severity of several diseases, such as myocardial infarction,^19^ pulmonary fibrosis,^20^ acute liver injury^21^ and osteoarthritis.^22^ The loss of Bach1 promoted mesendodermal differentiation of hESCs by activating the Wnt/β‐catenin signalling pathway.^14^ Further evidence shows that a Bach1 inhibitor could suppress osteoclastogenesis.^23^ Although the essential role of Bach1 in regulating the function and differentiation of stem cells has been demonstrated, the expression and functional mechanism of Bach1 in human PDLCs under inflammatory conditions is largely unknown.

Polycomb repressive complex 2 (PRC2) is a chromatin‐associated methyltransferase catalysing lysine 27 on histone H3 (H3K27) and inducing transcriptional repression of target genes.^24^, ^25^ EZH2 is the catalytic subunit of PRC2. The typical effect of EZH2 is gene silencing by catalysing trimethylation of lysine 27 of histone H3 (H3K27me3) in a PRC2‐dependent manner.^26^ EZH2 knockdown has been verified to inhibit the TLR4/MyD88/NF‐B signalling pathway to promote osteogenesis.^27^ Bach1 has been shown to interact with PRC2, manifested as Bach1, H3K27me3 and EZH2, having broad colocalization in the genome, inhibiting gene expression by recruiting PRC2 into the promoter of the gene.^14^ Current research on the effects of EZH2 and Bach1 interactions is still limited. The exact link between EZH2, Bach1 and osteogenesis under inflammatory conditions must be further explored. The regulation of osteoblastic genes by this binding effect and the specific mechanism by which Bach1 and EZH2 regulate the osteogenesis process were explored in this paper.

In this context, we hypothesized that inhibition of Bach1 would promote the osteogenesis of PDLCs under inflammatory conditions. Therefore, in the present study, we evaluated Bach1 expression in periodontitis tissue and PDLCs. Then, we investigated whether Bach1 knockdown could overcome oxidative stress and alleviate the reduction in osteogenesis caused by the inflammatory microenvironment. Moreover, we clarified the synergistic effect of EZH2 and Bach1 in regulating the expression of related genes. Our study is expected to reveal Bach1 as a potential target for cell‐based periodontal regeneration in periodontitis treatment.

## 2 MATERIALS AND METHODS

### 2.1 Collection of human periodontal ligament tissue

Human periodontal ligament tissues were harvested from PDL tissues of 20 donors. Among them, 10 extracted periodontal healthy, caries‐free third molars or premolars due to orthodontic reduction were collected as the healthy group. Ten teeth that could not be preserved due to periodontitis were collected as the periodontitis group. Immediately after the teeth were extracted, the teeth were rinsed with physiological saline to remove blood stains. The periodontal ligament tissue was then scraped with a periodontal scaler and finally stored in a 4% paraformaldehyde solution for subsequent immunohistochemical analysis. This work was approved by the Ethics Committee of Nanjing Stomatological Hospital, Medical School of Nanjing University (No. KY‐2020NL‐007).

### 2.2 Cell culture and osteogenic induction

The human PDLCs used in this study were purchased from ScienCell Research Laboratories (Catalog #2630, ScienCell) and were primary cells derived from human periodontal ligament tissues. PDLCs were cultured in complete Dulbecco's modified Eagle's medium (DMEM; Gibco) containing 10% foetal bovine serum (FBS; ScienCell) and 1% penicillin/streptomycin (HyClone). PDLCs between passages 2 and 5 were used for subsequent experiments. PDLCs were seeded in 6‐well plates and induced to osteogenic differentiation when they reached 60%–70% confluence. The medium was supplemented with osteo‐inductive medium, which was complete medium supplemented with 0.1 μM dexamethasone, 50 μg/mL ascorbic acid and 10 mM β‐glycerophosphate (Sigma–Aldrich).

Medium supplemented with TNF‐α (10 ng/mL) and IL‐1β (5 ng/mL) was used to simulate inflammatory conditions.

### 2.3 Lentivirus transfection

The lentiviral vector containing short hairpin RNA (shRNA) targeting Bach1 (sequence: TATGCACAGAAGATTCATAGG) was purchased from GeneChem. Co. (Shanghai, China), and scramble shRNA (sequence: TTCTCCGAACGTGTCACGT) served as a control. All lentiviral vectors contained green fluorescent protein (GFP). PDLCs in the logarithmic growth phase were digested and seeded into a six‐well plate at a density of 5 × 10^4^ per well. The lentivirus was diluted with DMEM without penicillin/streptomycin to prepare lentivirus suspensions of different concentrations, and the transfection reagent polybrene was added at a final concentration of 8 μg/mL to infect PDLCs for 12 h in an incubator at 37°C and 5% CO_2_. The proportion of GFP‐positive cells was observed by inverted fluorescence microscopy, and a positive rate greater than 95% was considered a suitable infection efficiency. Western blot and qRT–PCR were used to test the interference efficiency of Bach1 to determine the optimal multiplicity of infection (MOI) for lentivirus transfection.

### 2.4 ALP and Alizarin red S (ARS) staining

After 7‐ or 21‐day osteogenic induction, PDLCs were washed with PBS twice and then fixed with 4% paraformaldehyde for 30 min. Then, the cells were stained using an ALP staining kit (Beyotime) and ARS staining solution (Cyagen) according to the manufacturer's instructions.

### 2.5 Intracellular ROS Detection

The intracellular ROS levels in different groups of PDLCs were detected using a Reactive Oxygen Species Assay Kit (Beyotime, China). Briefly, the fluorescent probe DCFH‐DA was added to every group of PDLCs and incubated for 20 min after being diluted with serum‐free medium to a final concentration of 10 μmol/L. Then, the cells were washed and harvested, and the cell suspension was collected into flow tubes for centrifugation and washing. Finally, cell suspension samples were detected by flow cytometry to analyse the percentage of ROS‐positive cells.

### 2.6 Glutathione (GSH) detection

The intracellular GSH levels in different groups of PDLCs were detected using a reduced glutathione assay kit (Jiancheng Bioengineering Institute, China). Cell samples were cleaved by ultrasonication, and then reagents were added according to the instructions. The absorbance value was measured at a wavelength of 420 nm after the colour reaction.

### 2.7 Establishment of an experimental periodontitis model and periodontal defect model in rats

Animal experiments were carried out in accordance with ethical regulations and approved by the Laboratory Animal Welfare and Ethical Review of Nanjing University (No: IACUC‐2003053). Seven‐week‐old male SD rats were housed under specific‐pathogen free (SPF) conditions with a 12‐h light/dark cycle and adaptively fed for 1 week. Rats were anaesthetized with sodium pentobarbital by intraperitoneal injection, and all animal model construction experiments were carried out in an SPF animal operating room.

For the experimental periodontitis model establishment, five SD rats were subjected to ligation around the maxillary second molar on the left side with 4–0 medical sutures. The sutures were embedded under the gum and knotted buccally. Bilateral molars with no ligation served as controls. After 2 weeks, all rats were sacrificed by overdose with anaesthetics, and the bilateral maxillae were harvested for further histological analysis.

For the periodontal defect model establishment, 20 SD rats were maintained and anaesthetized under the same conditions as mentioned above and divided into four groups (5 rats per group) as follows: the cells transplanted into periodontal defects were shCtrl PDLCs (Ctrl group), shCtrl cultured with inflammatory medium (Ctrl+ Inflam group), shBach1 PDLCs (shBach1 group), and shBach1 PDLCs cultured with inflammatory medium (shBach1+ Inflam group). After the skin of the operation area was shaved and sterilized, a 2 cm incision was made from 5 mm away from the corner of the mouth parallel to the inferior border of the mandible. The masseter muscle and underlying periosteum were separated to expose the buccal alveolar bone of the first and second mandibular molars. Then, a periodontal defect of approximately 5 mm by the mesio‐distal direction and 2 mm by the crown‐root direction was made until reaching the root surfaces with a low‐speed grinder and spherical bur under physiological saline irrigation. The surface of the exposed root was curetted to remove the residual periodontal ligament and cementum. After that, 3 × 10^4^ PDLCs from every group were mixed into 20 μL human fibrinogen (2.5 mg/mL; Merck, Germany) solution, and the Inflam group was supplemented with 10 ng/mL TNF‐α and 5 ng/mL IL‐1β. Subsequently, 1 μL thrombin (25 U/mL; Merck, Germany) was added to catalyse solidification and implanted into the periodontal defect. Finally, a precut collagen membrane with a size of approximately 6 × 3 mm was used to cover the defect, and the wound was sutured (Figure S1). The rats were given an intraperitoneal injection of penicillin for three consecutive days to prevent wound infection. Three weeks after the operation, all the rats were sacrificed by an overdose of anaesthetics, and the mandible was harvested for further micro‐CT and histological analysis.

To evaluate the repair of periodontal defects, mandibles containing periodontal defect areas were scanned by micro‐CT (Hiscan XM Micro CT, Suzhou Hiscan Information Technology). The bone mineral content (BMC), trabecular thickness (Tb. Th), trabecular number (Tb. N), bone volume (BV), tissue volume (TV) and relative bone volume (BV/TV) of the newly formed bone in every group were analysed with Hiscan Analyzer software (Version 3.0, Suzhou Hiscan Information Technology).

### 2.8 Histological analysis

The periodontal ligament tissues were embedded in paraffin after fixation with 4% paraformaldehyde for 48 h, while the samples of rat jaws were embedded in paraffin after decalcification. The fixed jaws were placed into 10% EDTA decalcification solution for 2–3 months, and the decalcification solution was changed every 3 days until the bone tissue could be penetrated by a probe without obvious resistance. Then, the paraffin blocks were sliced to harvest thin sections (4 μm), which were stained with haematoxylin and eosin (H&E) and Masson staining for histological observation. The expression of Bach1 (antibody obtained from Bioworld, BS72938) in the periodontal tissues was detected by immunohistochemistry, and Bach1 expression was quantified by ImageJ software.

### 2.9 Quantitative real‐time polymerase chain reaction (qRT–PCR)

Total RNA was extracted using a FastPure® Cell/Tissue Total RNA Isolation Kit V2 (Vazyme, catalog #RC112‐01), and cDNA was prepared through reverse transcription using HiScript® III RT SuperMix for qPCR (Vazyme, catalog #R323‐01). The mRNA expression of different genes was analysed by real‐time PCR (ABIViiA7, Thermo Fisher Scientific), and the relative expression was calculated using the 2^−ΔΔCT^ method normalized to GAPDH. The primers used in this study are listed in Table S1.

### 2.10 Western blotting

The total proteins were extracted by RIPA buffer containing 1 mmol/L PMSF, separated on SDS–PAGE gels by electrophoresis and then transferred to PVDF membranes (Millipore, catalog #IPVH00010, IRL). Subsequently, the membranes were blocked in 5% BSA buffer for 1 h at room temperature and incubated with anti‐Bach1(Santa cruz, catalog #sc‐271211), anti‐BMP‐6 (Santa cruz, catalog #sc‐57042), anti‐CAP (Santa cruz, catalog #sc‐53947), anti‐COL I (Proteintech, catalog #14695‐1‐AP), anti‐OPN (Proteintech, catalog #22952‐1‐AP), anti‐RUNX2 (Proteintech, catalog #20700‐1‐AP), anti‐OPG (Proteintech, catalog #40938), anti‐HO‐1 (Proteintech, catalog #10701‐1‐AP), anti‐GCLM (Proteintech, catalog #14241‐1‐AP), anti‐EZH2 (Proteintech, catalog #21800‐1‐AP), anti‐β‐tublin (Proteintech, catalog #10094‐1‐AP), anti‐β‐actin (Proteintech, catalog #66009‐1‐Ig) and anti‐GAPDH (Proteintech, catalog #60004‐1‐Ig) overnight at 4°C, followed by incubation with secondary antibodies (Biogot technology, anti‐rabbit: catalog #BS13278; anti‐mouse: catalog #BS12478). Finally, the target protein bands were visualized by ECL luminescence reagents (NCM Biotech, catalog #10300) and an imaging system.

### 2.11 Immunoprecipitation

PDLCs under control or inflammatory conditions were collected and lysed to extract total protein. A small part of the harvested cell lysates was used for western blotting, and the rest of the lysates were incubated with 4 μg EZH2 antibody (Proteintech) or Bach1 antibody at 4°C overnight and precipitated with Protein A/G Magnetic Beads (Thermo Fisher Scientific) for another 1 h at room temperature. The beads were washed 4 times with washing buffer (PBS buffer with 0.05% Tween 20) on the magnetic stand, and the immunoprecipitated proteins were boiled for 10 min in loading buffer. Finally, the immunoprecipitated proteins were detected by Western blotting.

### 2.12 Chromatin immunoprecipitation (ChIP)

The DNA and protein in cells were cross‐linked using 1% formaldehyde solution under physiological conditions. Chromatin was broken up by ultrasonic waves, and then, anti‐EZH2 antibody, anti‐H3K27me3 antibody or IgG antibody was added to precipitate cross‐linked complexes. DNA fragments that bind to EZH2, H3K27me3 or IgG were precipitated. Decrosslinking was performed, the protein was removed with protease, and the DNA fragment was purified. DNA sequences specifically combined with EZH2, H3K27me3 or IgG were screened by qRT–PCR. The primers used in this study are listed in Table S2. During the data analysis, the IgG binding amount was subtracted from the EZH2 or H3K27me3 binding amount to obtain the relative amount.

### 2.13 Fluorescence colocalization analysis

PDLCs were seeded on multi‐coverslips overnight. The cells were washed with PBS and fixed with 4% paraformaldehyde in PBS for 30 min at room temperature, and cells were followed treated with 1% Triton X‐100. After blocking with 5% bovine serum albumin in PBS at room temperature for 1 h, these cells were incubated with rabbit anti‐Bach1 antibody and Alexa Fluor 647‐conjugated goat anti‐rabbit IgG (Thermo Fisher Scientific, catalog # A‐21246). After washing, coverslips were incubated with rabbit anti‐EZH2 antibody and Alexa Fluor 488‐conjugated goat anti‐rabbit IgG (Thermo Fisher Scientific, catalog # A‐11034). The stained PDLCs were analysed using Zeiss LSM880 laser scanning fluorescence microscope system.

### 2.14 Statistical analysis

GraphPad Prism 8.0 was used to analyse the experimental data, and the experimental results are expressed as the mean ± standard deviation. Independent sample t‐tests were used to compare the differences between the two groups, and one‐way analysis of variance was used to compare the differences between multiple groups. When the *p* value <0.05, a significant difference was considered.

## 3 RESULTS

### 3.1 Bach1 expression in periodontal tissues with periodontitis and PDLCs under inflammatory conditions

**FIGURE 1:**
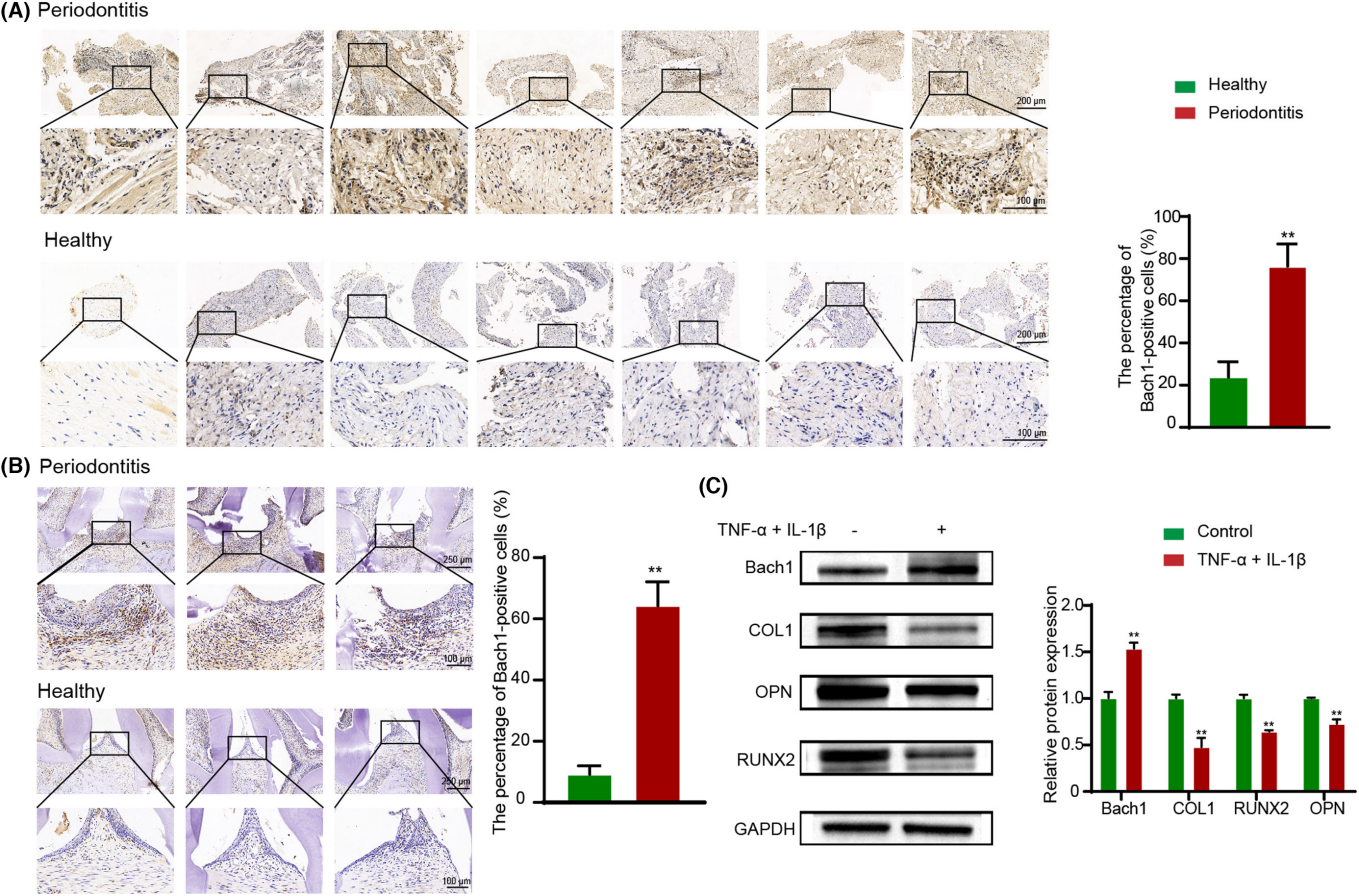
Bach1 expression in periodontal tissues with or without periodontitis and PDLCs under normal or inflammatory conditions. (A) Immunohistochemistry analysis of Bach1 expression in human periodontal tissues of healthy teeth and teeth with periodontitis. (B) Immunohistochemistry analysis of Bach1 expression in periodontal tissues of teeth with experimental periodontitis and healthy self‐control rats. (C) Western blotting of Bach1 and osteoblast differentiation‐related protein (RUNX2, OPN, COL1) expression under normal (osteo‐inductive culture medium) or inflammatory (osteo‐inductive culture medium supplemented with TNF‐α and IL‐1β) conditions. Data are presented as the mean ± SD, **p < 0.01.

Bach1 expression in human periodontal tissues of healthy teeth and teeth with periodontitis was evaluated by immunohistochemistry. The percentage of Bach1‐positive cells was significantly higher in periodontal tissues with periodontitis than in the healthy group (*p* < 0.01, Figure 1A).

Given that the ages of donors between the healthy and periodontitis groups could not be matched in human tissues, we also detected Bach1 expression in an experimental periodontitis model in rats, and contralateral periodontal tissue served as a healthy self‐control. The results of Bach1 immunohistochemistry staining indicated the same conclusion in the periodontal tissues of rats (Figure 1B).

To further investigate the role of Bach1 under inflammatory conditions, we established a highly reproducible model of PDLC inflammation and impaired osteogenesis with 10 ng/mL TNF‐α and 5 ng/mL IL‐1β. The PDLCs expressed osteoblast differentiation‐related proteins such as RUNX2, OPN and COL1 under osteo‐inductive conditions, and after 7 days of induction with inflammatory factors, the decreased protein expression of RUNX2, OPN and COL1 was confirmed by western blotting (*p* < 0.01). Meanwhile, we observed a significant elevation in Bach1 protein expression in PDLCs in response to inflammatory culture conditions (*p* < 0.01, Figure 1C).

In general, we evaluated Bach1 expression in PDLCs and periodontal samples from humans and rats and confirmed that Bach1 expression was significantly increased under inflammatory conditions. Meanwhile, the osteogenic ability of PDLCs was inhibited under inflammatory conditions. Therefore, we speculated that Bach1 may be involved in the inhibitory effect of inflammatory conditions on osteogenesis.

### 3.2 Bach1 knockdown rescues the inflammation‐induced reduction in osteogenesis

To investigate the potential function of Bach1 in PDLCs under inflammatory conditions, we constructed a lentiviral vector containing shRNA targeting BACH1 to stably downregulate Bach1 (shBach1) in PDLCs. Meanwhile, PDLCs transfected with a lentiviral vector targeting the scramble sequence served as a control (shCtrl). According to qRT–PCR analysis, the Bach1 interference efficiency of the shBach1 group was 60% at MOI = 50 compared with the shCtrl group. Then, western blotting analysis confirmed the interference efficiency (Figure S2). We observed osteogenic differentiation of shBach1 and shCtrl cells under normal or inflammatory conditions.

**FIGURE 2:**
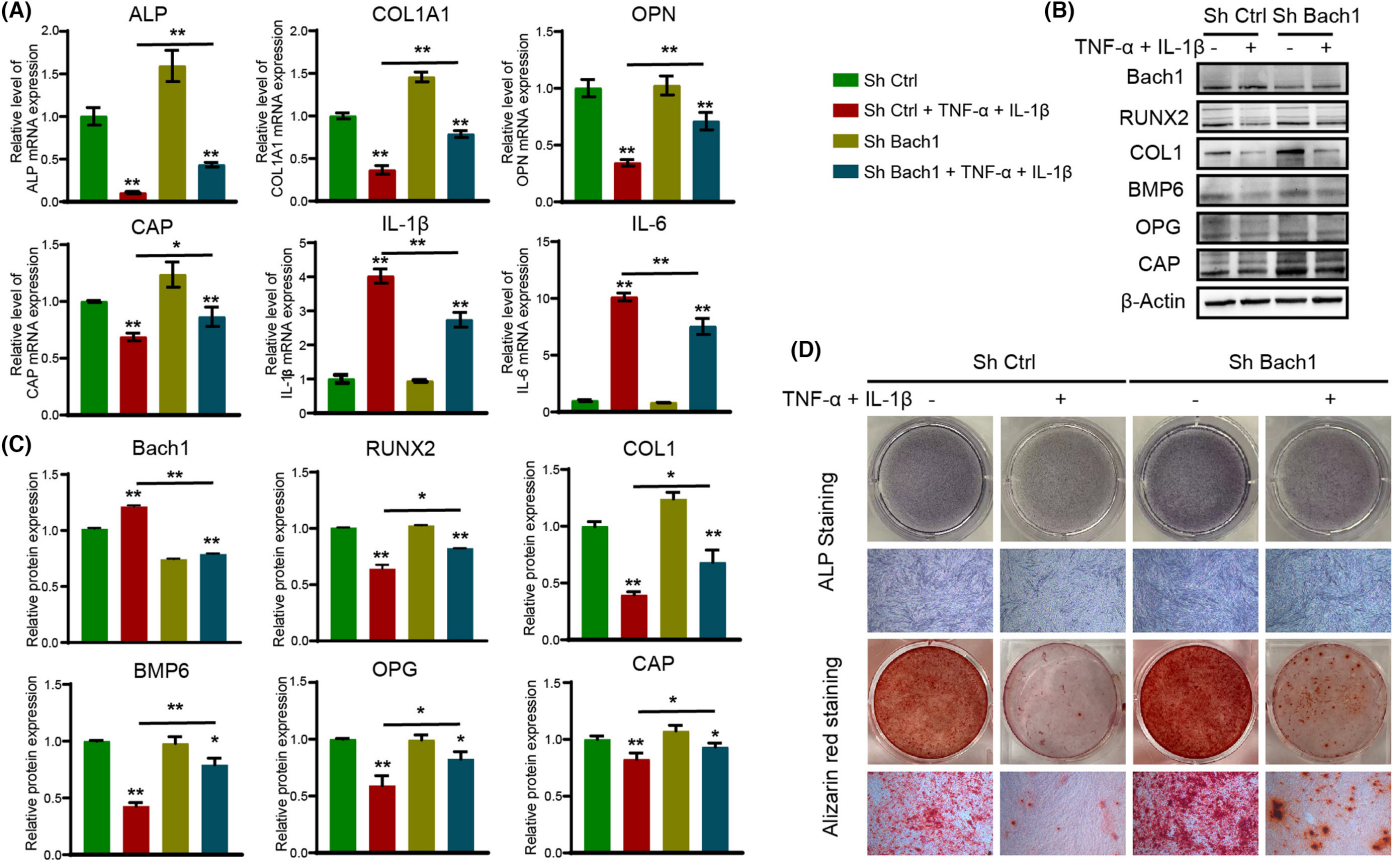
Bach1 knockdown rescues inflammatory damage in osteogenesis. (A) qRT–PCR analysis of ALP, COL1A1, CAP, IL‐1β and IL‐6 mRNA expression in shCtrl and shBach1 PDLCs under normal or inflammatory conditions after 7 days of osteogenic induction. (B, C) western blotting and quantitative analysis of Bach1, RUNX2, COL1, BMP6, OPG and CAP protein expression in shCtrl and shBach1 PDLCs under normal or inflammatory conditions after 7 days of osteogenic induction. (D) ALP and Alizarin red staining of shCtrl and shBach1 PDLCs under normal or inflammatory conditions. Data are presented as the mean ± SD, *p < 0.05, **p < 0.01.

According to qRT–PCR analysis, the expression levels of osteogenic genes (ALP, COL1A1) and the cementogenesis gene CAP were decreased, while inflammation‐related genes (IL‐1β, IL‐6) were significantly increased in response to inflammatory factors. In contrast, the knockdown of Bach1 rescued the effect of inflammatory conditions on the osteogenic differentiation and inflammation‐related gene expression of PDLCs (Figure 2A).

Similarly, western blot analysis confirmed that the expression of osteoblast differentiation‐related proteins (RUNX2, COL1, BMP6, OPG) and the cementogenesis‐specific protein CAP was significantly decreased under inflammatory conditions, while shBach1 PDLCs showed unchanged BMP6, OPG and RUNX2 protein expression but increased COL1 and CAP protein expression compared to shCtrl under normal conditions. In the presence of inflammatory factors, shBach1 PDLCs showed increased BMP6, OPG and RUNX2 protein expression compared to shCtrl PDLCs, indicating that knockdown of Bach1 offset the inhibitory effect of inflammatory conditions on BMP6, OPG and RUNX2 protein expression (Figure 2B,C).

After 7 or 21 days of osteogenic induction, ALP or Alizarin red staining was performed, and the results indicated that osteogenic differentiation was largely impaired in both shBach1 and shCtrl cells in response to inflammatory conditions, whereas Bach1 knockdown rescued the inflammation‐induced reduction in osteogenesis (Figure 2D). Interestingly, we observed that shBach1‐modified PDLCs exhibited enhanced osteogenic differentiation to a certain extent under normal conditions in vitro, suggesting that Bach1 not only rescued the effect of inflammatory conditions on osteogenesis but could also play an important role in the osteogenic differentiation of PDLCs under normal conditions.

In summary, these data suggested that inflammatory factor treatment resulted in a significant injury in the osteogenesis of either shBach1 or shCtrl, but the injury was rescued in shBach1 compared with shCtrl.

### 3.3 Bach1 knockdown alleviated the inflammation‐induced oxidative stress level

**FIGURE 3:**
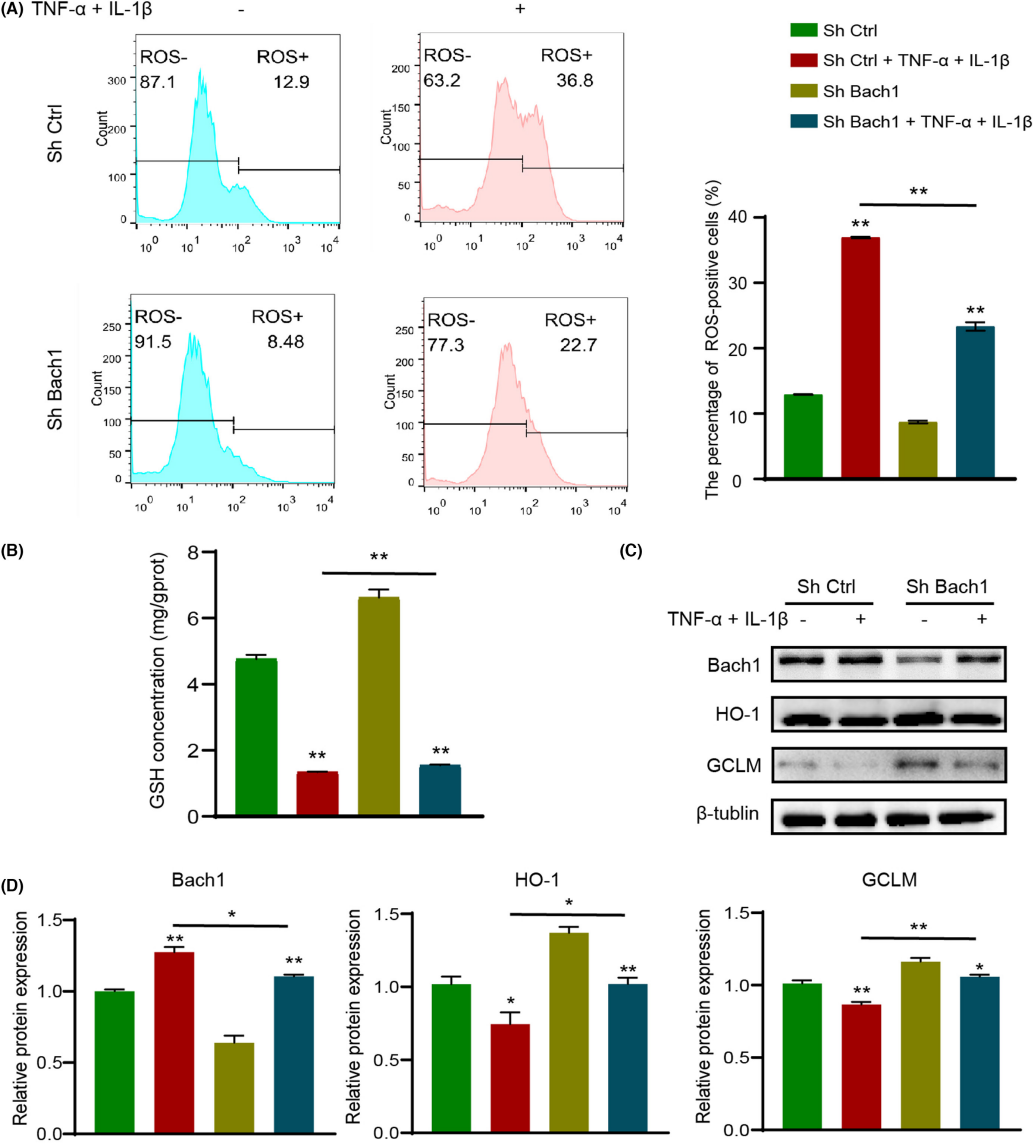
Bach1 knockdown alleviated inflammation‐mediated oxidative stress. (A) ROS production of shBach1 and shCtrl under normal (normal α‐MEM culture medium) or inflammatory (medium supplemented with TNF‐α and IL‐1β) conditions. (B) Determination of GSH content. (C) Protein expression of Bach1, HO‐1 and GCLM. (D) Quantitative analysis of WB results. Data are presented as the mean ± SD, *p < 0.05, **p < 0.01.

It is known that inflammatory conditions can induce an increased oxidative stress level. Considering that Bach1 was reported to be involved in the oxidative stress response in other disease models, we further explored whether Bach1 knockdown could inhibit the inflammation‐mediated oxidative stress that led to inflammatory damage in osteogenesis. We detected ROS production in shBach1 and shCtrl cells under normal or inflammatory conditions and observed that the percentage of ROS‐positive PDLCs was significantly increased under inflammatory conditions and decreased after Bach1 knockdown. The results indicated that the shBach1 group was more resistant to inflammatory conditions than the shCtrl group (Figure 3A). GSH is a critical antioxidant within cells, preventing cell damage from reactive oxygen species such as free radicals and peroxides. Measurement of GSH expression in PDLCs also confirmed increased resistance to oxidative stress after knockdown of Bach1 (Figure 3B). The expression of HO‐1 and GCLM was reduced under inflammation, but the proportion of inhibition was reduced in shBach1 (Figure 3C,D). The results above proved that Bach1 knockdown alleviated the inflammation‐induced oxidative stress level.

### 3.4 Knockdown of Bach1 made PDLCs more resistant to inflammatory conditions to repair periodontal defects in vivo

**FIGURE 4:**
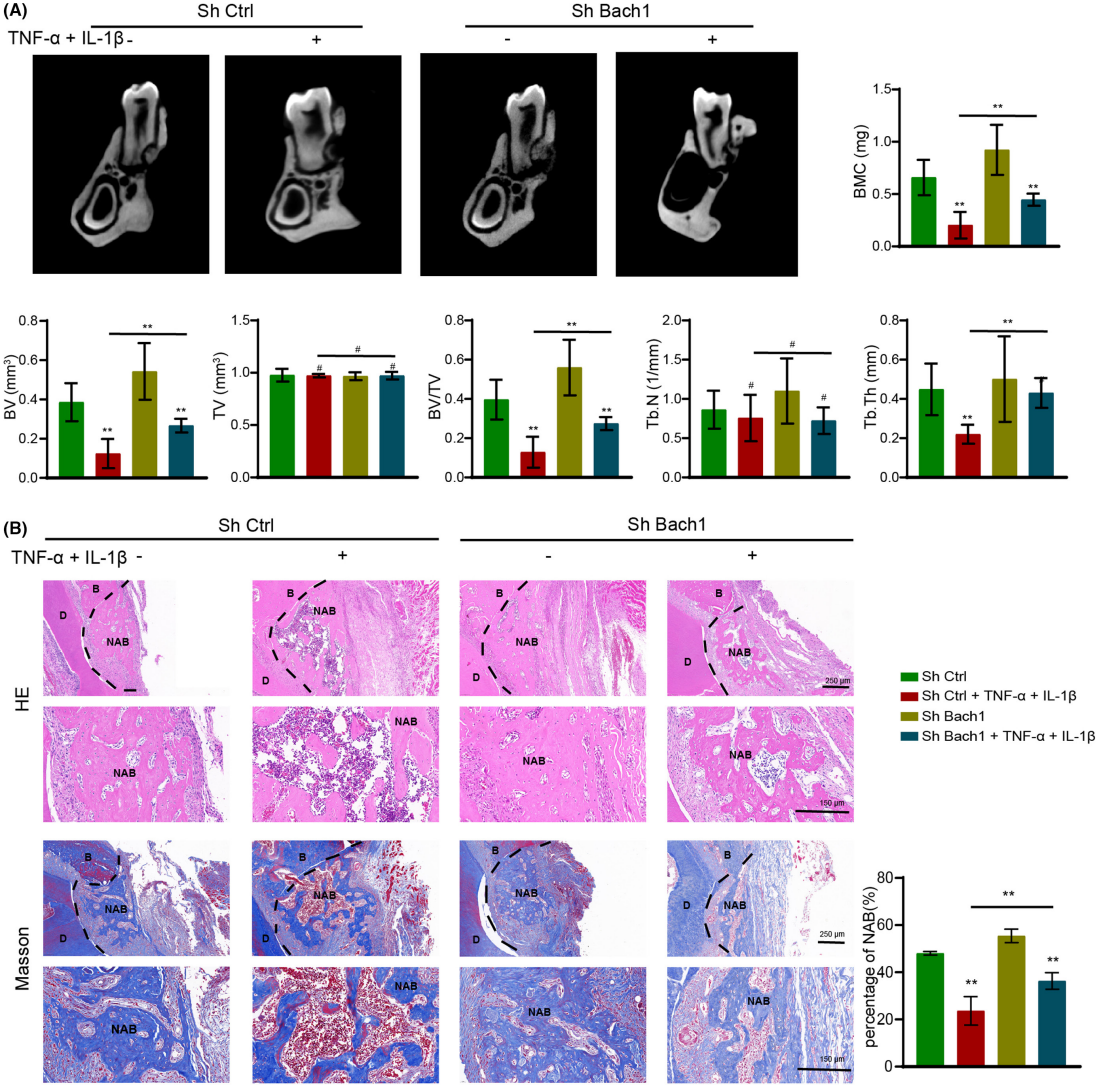
Knockdown of Bach1 protected PDLCs from inflammatory damage to repair periodontal defects in vivo. (A) ShCtrl and shBach1 PDLCs cultured under normal or inflammatory conditions were transplanted into the rat periodontal defect model, and defect repair was evaluated after 3 weeks. MicroCT scanning images of the mandibular first molars and analysis of bone‐related parameters (BV, TV, BV/TV, Tb. Th, Tb. N and BMC). (B) H&E and Masson staining images and quantification of NAB (D indicates dentin; NAB indicates newly formed alveolar bones). Data are presented as the mean ± SD, *p < 0.05, **p < 0.01, # no significant difference.

To ascertain Bach1‐regulated osteogenesis under inflammatory conditions in vivo, we transplanted PDLCs from every group into the rat periodontal defect model and evaluated defect repair after 3 weeks. Newly formed bone in the periodontal defect was analysed with microCT scanning. There was no significant difference in TV and Tb. Th in every group. The BV, BV/TV, Tb. N and the BMC of the newly formed bone were decreased in the Inflam group. When PDLCs were cultured under inflammatory conditions, shBach1 (shBach1 + Inflam group) showed better bone regeneration capacity than shCtrl (shCtrl+Inflam group), as well as increased BV, BV/TV, Tb. N and BMC (Figure 4A). Then, we evaluated the amount of the newly formed tissue in each group by H&E and Masson staining. Similar to the microCT results, the amount of newly formed alveolar bone (NAB) was significantly decreased in the Inflam group, but shBach1 rescued this inflammatory damage compared to shCtrl (Figure 4B). Overall, these results suggested that downregulation of Bach1 protects PDLCs from inflammatory damage in bone regeneration capacity in vivo.

### 3.5 Bach1 synergizes with EZH2 to repress the expression of related genes

**FIGURE 5:**
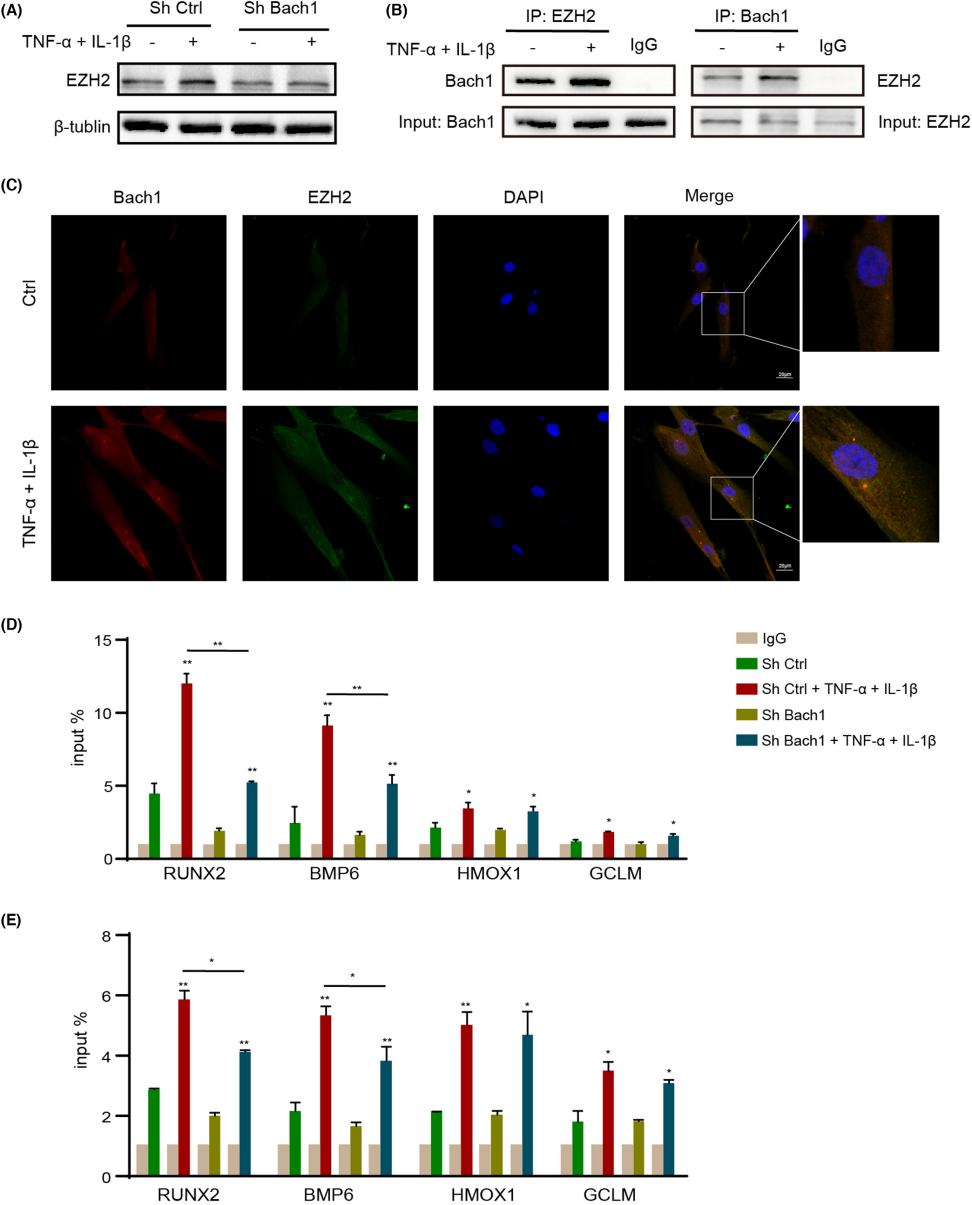
Bach1 interacts with EZH2 to repress the expression of related genes. (A) Expression of EZH2 PDLCs in normal or inflammatory culture was analysed by Western blotting. (B) The interaction of EZH2 with Bach1 was investigated by immunoprecipitation analysis. (C) Colocalization of Bach1 and EZH2. (D) The binding of the promoters of the RUNX2, BMP6, HMOX1 and GCLM genes with EZH2 was investigated by ChIP–qPCR. (E) The binding of the promoters of the RUNX2, BMP6, HMOX1 and GCLM genes with H3K27me3 was investigated by ChIP–qPCR. Data are presented as the mean ± SD, *p < 0.05, **p < 0.01.

The expression of EZH2 was slightly increased under inflammatory stimulation, but knockdown of Bach1 had no effect on its expression (Figure 5A). Previous studies have verified that EZH2 binds to Bach1 in human embryonic stem cells (hESCs).^14^ That combination was also confirmed in PDLCs by immunoprecipitation, and we further found that the binding of Bach1 and EZH2 was increased under inflammatory conditions (Figure 5B). Consistently, result of immunofluorescence colocalization verified the increase in the expression of Bach1 and EZH2 and the colocalization signals in inflammatory environments (Figure 5C). Therefore, we speculated that the increased binding of Bach1 and EZH2 under inflammatory conditions could promote the exercise of gene regulatory function. ChIP experiments confirmed that EZH2 could bind to the promoter regions of RUNX2, BMP6, HMOX1 and GCLM. Compared with RUNX2 and BMP6, the binding of HMOX1 and GCLM to EZH2 was lower. In inflammatory conditions, EZH2 significantly bound to its target promoter regions of RUNX2 and BMP6, and the binding was reduced after knockdown of Bach1, while that was not reflected in HMOX1 and GCLM (Figure 5D). The H3K27me3 CHIP experiment also verifies the above results. In inflammatory conditions, H3K27me3 bound to the target promoter regions of RUNX2 and BMP6, and the binding was decreased after knockdown of Bach1, while that was not reflected in HMOX1 and GCLM (Figure 5E). HMOX1 and GCLM are the target genes of Bach1,^18^ so the transcriptional regulation of Bach1 is dominant. The above results proved that EZH2 binds to Bach1 and dominates the promoter regions of RUNX2 and BMP6 to regulate osteogenesis under inflammatory conditions.

**FIGURE 6:**
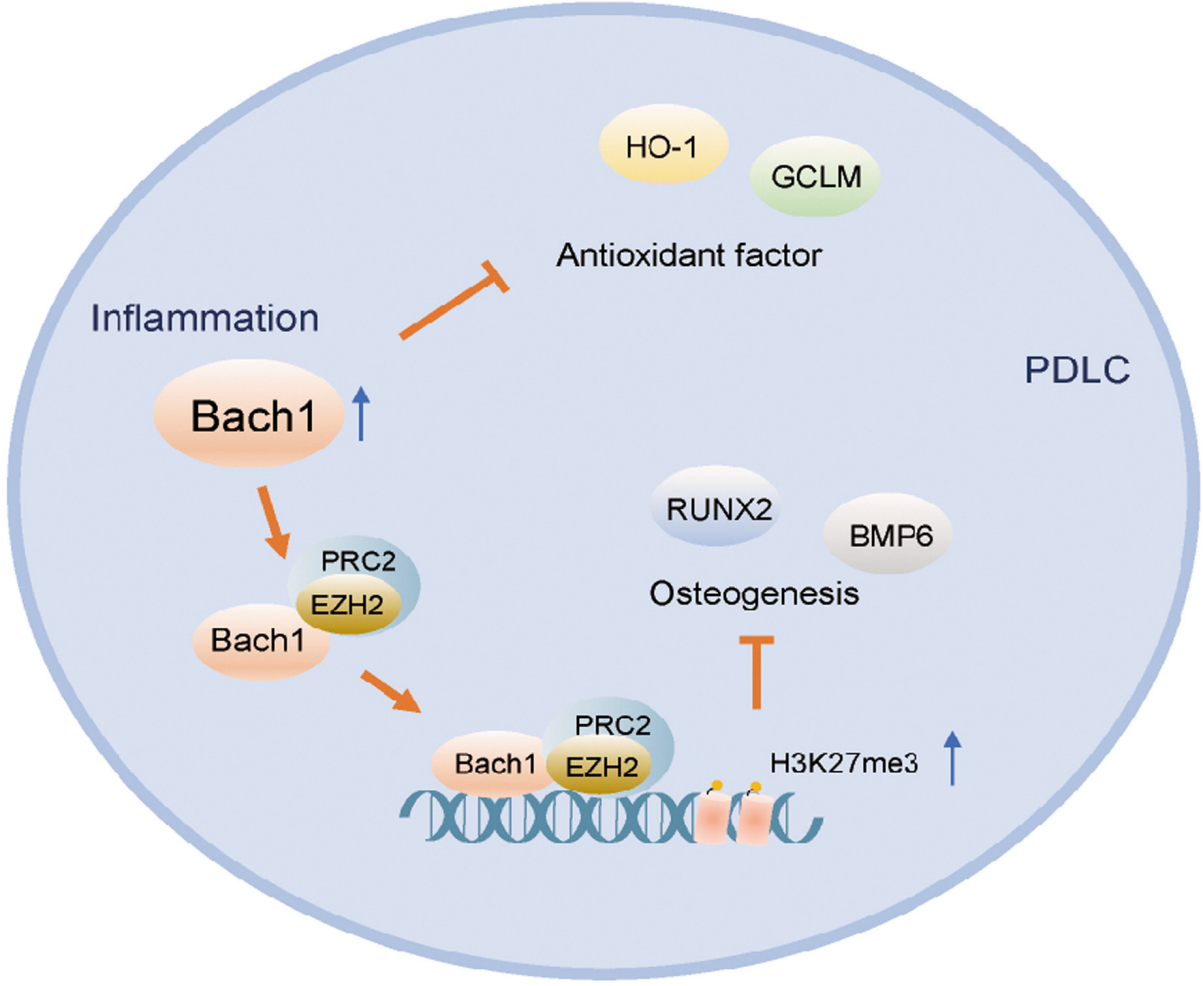
Graphical summary. The increased Bach1 expression in PDLCs under inflammatory conditions directly inhibits antioxidant factors such as HO‐1 and GCLM. Meanwhile, Bach1 interacts with the histone methyltransferase EZH2, and its binding is increased under inflammatory conditions. Bach1 enhanced the ability of EZH2 to catalyse H3K27me3 on the promoter region of RUNX2 and BMP6, thus repressing the gene expression of osteoblastic genes. Knockdown of Bach1 protects PDLCs from inflammatory damage to periodontal bone regeneration.

Accordingly, on the one hand, the expression of Bach1 was elevated in response to inflammation, which inhibited the antioxidant effect of PDLCs by directly repressing the expression of HO‐1 and GCLM. On the other hand, Bach1 interacted with EZH2 and enhanced the ability of EZH2 to catalyse histone methylation of RUNX2 and BMP6, thus inhibiting the expression of osteoblastic genes. Therefore, knockdown of Bach1 significantly alleviated oxidative stress and promoted osteogenesis under inflammatory conditions (Figure 6).

## 4 DISCUSSION

In the development of periodontitis, when periodontal tissues are exposed to pathogens and inflammatory conditions, ROS are produced to kill bacteria, but excess ROS accumulation damages PDLCs and tissues, leading to a decreased regenerative repair ability. Oxidative stress is one of the important factors involved in the pathogenesis of periodontitis and alveolar bone loss. Bach1 is an important transcription factor involved in regulating oxidative stress that competes with Nrf2 and negatively regulates antioxidant response elements (AREs).^28^ Wang et al. found that Bach1 regulated genes participating in the cell cycle and oxidative stress response, induced ROS production, and then promoted cell apoptosis in endothelial cells.^29^ Receptor activator of nuclear factor‐κB ligand (RANKL) induces Nrf2 nuclear export and Bach1 nuclear import, thereby augmenting ROS accumulation and osteoclastogenesis.^30^ In this study, our results indicated that the expression level of the oxidative stress‐related factor Bach1 was significantly increased in periodontal tissues of periodontitis. The differences in Bach1 expression between periodontitis and healthy periodontal tissues were proven in human periodontal ligament tissue, the periodontium of rats, and PDLCs under inflammatory and normal conditions. Consistent with our results, Pomatto et al. reported that cytosolic and nuclear Bach1 expression was both heightened when cells were subjected to chronic hyperoxia conditions, and Bach1 exhibited nuclear accumulation after cells were treated with H_2_O_2_ stimulation for 18 h.^31^ In another experiment, an acute liver injury rat model was developed by intravenous LPS administration, and nuclear Bach1 expression was decreased transiently and then quickly recovered after LPS treatment.^21^ Similarly, mice were subjected to bleomycin administration to develop pulmonary fibrosis that suffered oxidative injury, and Bach1 expression in the lung tissue was significantly increased.^32^ Collectively, these findings indicated that Bach1 was induced and might be an important regulator in the pathogenesis of periodontitis.

Bach1 is a transcriptional inhibitor of many antioxidant genes. Cumulative evidence verified that inhibition of Bach1 possessed anti‐inflammatory and antioxidative stress functions. The Bach1 inhibitors HPP‐A, HPP‐B, HPP‐C and HPP‐E were reported to reduce intracellular ROS levels and attenuate RANKL‐mediated osteoclastogenesis, which has the potential to treat destructive bone diseases.^23^ In a pulmonary fibrosis mouse model, knockdown of Bach1 reduced oxidative injury and inflammation in lung tissues and cells and ameliorated lung fibrosis by blocking the Erk pathway.^20^ In accordance with these findings, our study revealed that inflammatory conditions induced increased oxidative stress and reduced osteogenic ability, while knockdown of Bach1 alleviated inflammatory damage. Tian et al. reported that Bach1 inhibition alleviated osteoblast apoptosis, improved cell viability and reduced ROS production.^33^ Induction of Bach1 nuclear export increased Nrf2 nuclear import and attenuated intracellular ROS signalling, thus diminishing osteoclastogenesis.^30^ These studies showed that inhibition of Bach1 reduced bone destruction and promoted bone regeneration. However, studies on muscle regeneration have shown the reverse.^34^ The reason may be that oxidative stress may accelerate muscle repair, which is different from periodontal tissue regeneration.^35^ Our experiments showed that shBach1‐modified PDLCs exhibited enhanced osteogenic differentiation and periodontal tissue regeneration to a certain extent. Although inflammatory factors other than TNF‐α and IL‐1β could induce the expression of Bach1 of other cells, such as macrophagocyte, PDLCs after Bach1 knockdown showed better osteogenesis in both inflammatory and non‐inflammatory settings. Further studies using Bach1 knockout animals are warranted to verify these results.

Furthermore, we demonstrated the binding of Bach1 and EZH2 in PDLCs and their regulatory role in osteoblastic gene expression under inflammatory conditions. EZH2 is a histone methyltransferase and alters downstream target gene expression by trimethylation of Lys‐27 in histone 3 (H3K27me3).^36^ It was reported that EZH2 could interact with Bach1.^14^ We verified the binding of Bach1 and EZH2 in PDLCs and found that the binding was increased under inflammatory conditions. HMOX1, GCLM and COL1A1 are known to be directly repressed by Bach1.^18^ Our results also confirmed that the expression of HO‐1, GCLM and COL1 was increased after knockdown of Bach1, which was consistent with previous studies. However, Bach1 could regulate the expression of the osteoblastic genes BMP6, OPG and RUNX2 under inflammatory conditions, but the regulation does not seem obvious under normal conditions, suggesting that Bach1 does not directly regulate osteogenic genes. According to bioinformatics prediction, EZH2 and Bach1 were both enriched in the promoter regions of these genes. Therefore, we further explored whether EZH2 was involved in the indirect regulation of osteoblastic genes by Bach1. The binding of EZH2 to the target promoter of RUNX2 and BMP6 was increased in response to inflammatory conditions but reduced after knockdown of Bach1. Bach1 enhanced the ability of EZH2 to catalyse H3K27me3 on the promoter region of RUNX2 and BMP6, thus repressing the gene expression of osteoblastic genes. Oxidative‐related genes, such as HMOX1 and GCLM, were directly regulated by Bach1 but not EZH2. The direct and indirect regulation of Bach1 jointly inhibited the antioxidative stress and osteogenesis of PDLCs under inflammatory conditions.

Therefore, Bach1 could be a promising target for enhancing periodontal tissue regeneration under periodontitis conditions. It has also been reported that drugs and biomaterials such as hyperoside, gold nanoparticles and sodium arsenite could elicit Bach1 nuclear export and increase the expression of antioxidative enzymes.^37^, ^38^, ^39^ At the genetic level, it has been reported that the elevated expression of ST8SIA1, a periodontitis risk gene, caused by weakening of Bach1's binding to effect T allele of its coinherited variants is a genetic cause of periodontitis.^40^ Further studies will be necessary to verify the role of these drugs in the treatment of periodontitis and the promotion of periodontal tissue regeneration.

## AUTHOR CONTRIBUTIONS

**Zhiyao Yuan:** Conceptualization (equal); writing – original draft (equal). **Junjie Li:** Data curation (equal); resources (equal). **Xihong Zou:** Data curation (equal); resources (equal). **Chaoyi Liu:** Methodology (equal); resources (equal); validation (equal). **Jiangyue Lu:** Formal analysis (equal); software (equal); visualization (equal). **Can Ni:** Methodology (equal); resources (equal). **Lai Tang:** Investigation (equal); resources (equal). **Xudong Wu:** Project administration (equal); writing – review and editing (equal). **Fuhua Yan:** Project administration (equal); writing – review and editing (equal).

## CONFLICT OF INTEREST STATEMENT

The authors declare no competing interests.

## Supporting information


Data S1.

## Data Availability

The data used to support the findings of this study are available from the corresponding author upon request. The data that support the findings of this study are available in the supplementary material of this article.
