# Supplementary material for: Knockdown of Bach1 protects periodontal bone regeneration from inflammatory damage
Source: J Cell Mol Med. 2023 Aug 21;27(22):3465–77. doi: 10.1111/jcmm.17916 (PMC10660620; doi:10.1111/jcmm.17916)
Supplement: Supplementary file 1 — Data S1. [file JCMM-27-3465-s001.docx]

**Supplementary Materials**


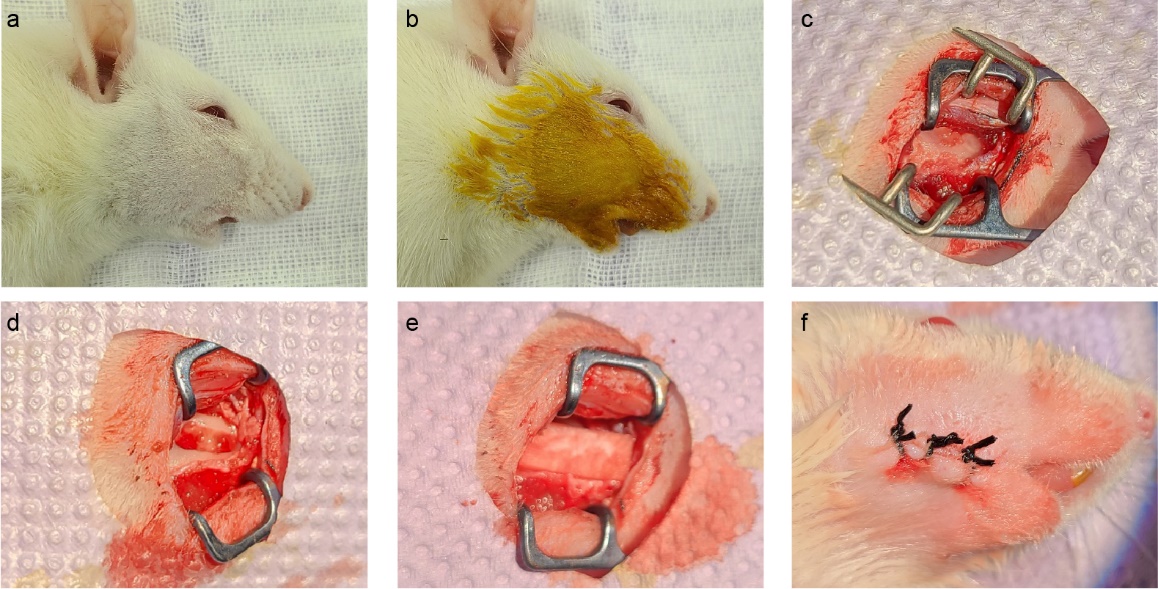


Supplementary Figure 1. Establishment of periodontal defect model in rats. (a) Anesthesia and shaving. (b) Sterilization of the surgical area. (c) Exposure of the buccal mandibular surface. (d) Preparation of the periodontal defect. (e) Transplantation of cells into the periodontal defect and coverage with collagen membrane. (f) Suture of the surgical wound.


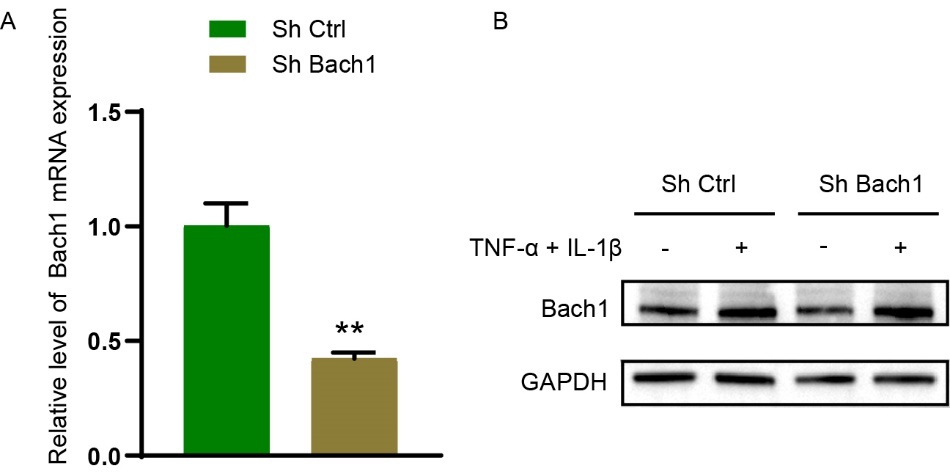


Supplementary Figure 2. The interference efficiency of shBach1. (a) qRT‒PCR analysis of Bach1 mRNA in shCtrl and shBach1 PDLCs. (b) Western blotting analysis of Bach1 protein expression in shCtrl and shBach1 PDLCs under normal or inflammatory conditions. Data are presented as the mean± SD, **p < 0.01.

**Supplementary tables**

Supplementary table 1. Primer sequences used in qRT‒PCR

| Gene | Forward Primer sequences | Reverse Primer sequences |
| --- | --- | --- |
| Bach1 | TCATTAGCTTGGGAGACGAC | TGCATTTTCAACAAGGACTG |
| CAP | CTGCGCGCTGCACATGG | GCGATGTCGTAGAAGGTGAGCC |
| COL-1 | AGAACAGCGTGGCCT | TCCGGTGTGACTCGT |
| OPN | CCAGCCAAGGACCAACTACA | CCAAGTGGCTACAGCATCTGA |
| ALP | GACCCTTGACCCCCACAAT | GCTCGTACTGCATGTCCCCT |
| IL-6 | AACAACCTGAACCTTCCAAAGA | TCAAACTCCAAAAGACCAGTGA |
| IL-1β | TTCCTGTTGTCTACACCAATGC | CGGGCTTTAAGTGAGTAGGAGA |
| GAPDH | CGCTCTCTGCTCCTCCTGTT | CCATGGTGTCTGAGCGATGT |

Supplementary table 2. Primer sequences used in ChIP‒qPCR

| Promoter of gene | Forward Primer sequences | Reverse Primer sequences |
| --- | --- | --- |
| BMP6-1  BMP6-2 | AAGACAGGGGACGCCTTTTC  GTGGGTCACCCCTCGATTTT | GGGGTCGGATGTGTTGACTT  TCTACCTACGAGGGCAGTGG |
| RUNX2-1 | TCCAAATCCTCATGAGTCACAA | CTTAAGTAAAGTGGGACTGCCT |
| RUNX2-2 | AGAACAGCGTGGCCT | TCCGGTGTGACTCGT |
| HMOX1-1 | CCAGCCAAGGACCAACTACA | CCAAGTGGCTACAGCATCTGA |
| HMOX1-2 | GACCCTTGACCCCCACAAT | GCTCGTACTGCATGTCCCCT |
| GCLM -1 | AACAACCTGAACCTTCCAAAGA | TCAAACTCCAAAAGACCAGTGA |
| GCLM -2 | TTCCTGTTGTCTACACCAATGC | CGGGCTTTAAGTGAGTAGGAGA |
